# Giant Precordial T Wave Inversion in a Patient with Gastroenteritis

**DOI:** 10.1155/2011/942045

**Published:** 2011-09-12

**Authors:** David Rott, David Leibowitz, A. Teddy Weiss

**Affiliations:** Department of Medicine, Hadassah Hebrew University Medical Center, Mt. Scopus, P.O. Box 24035, Jerusalem 91240, Israel

## Abstract

Giant precordial T wave inversion (GPTI) on ECG may be the result of several pathologies, including myocardial ischemia, pulmonary edema, pulmonary embolism, subarachnoid hemorrhage, apical hypertrophy, and postpacing. We describe a case of a 75-year-old woman who developed GPTI after an episode of gastroenteritis. To our knowledge, this is the first report of this ECG pattern associated with gastroenteritis.

## 1. Introduction

The appearance of giant precordial T wave inversion (GPTI) on an electrocardiogram (ECG) is alarming since it may represent anterior wall ischemia in patients presenting with acute coronary syndrome (ACS) [1]. Although GPTI is a more specific sign for ischemia as compared to other types of T wave inversion, it is definitely not pathognomonic and may be the result of other, nonischemic pathologies.

The present paper describes a patient with gastroenteritis and GPTI on ECG. 

## 2. Case Presentation

A 75-year-old woman with type II diabetes and hypertension presented to our hospital with dizziness, abdominal pain, nausea, and vomiting. Symptoms started 5 hours prior to presentation. She denied chest pain or shortness of breath. 

She had similar symptoms of abdominal pain, nausea, and vomiting 10 years prior to admission which required hospitalization. Her ECG then revealed symmetrical GPTI. Cardiac enzymes were normal. She was diagnosed with ACS; however, she refused to undergo coronary angiography. 

On current presentation pulse was 80 beats per minute, blood pressure 124/70 mm Hg, and the patient was afebrile. On physical examination she was in good general condition, lungs were clear, heart sounds were normal, and she had no cardiac murmurs. Abdomen was soft and nontender, neurological exam was unremarkable. 

Blood tests showed white blood count 7.6 per microliter, hemoglobin 14.7 gr%, platelets 202,000 per microliter, sodium 137 mmol/L, potassium 3.6 mmol/L, urea 6.7 mmol/L, creatinine 59 micromol/L, calcium 2.14 mmol/L, phosphate 1.0 mmol/L, albumin 38 gr/L, CPK 85 u/L, troponin T < 0.01 ng/mL, ALT 10 U/L, AST 28 U/L, Alk. P 60 U/L, T. Bil 5 micromol/L, and amylase 62 U/L.

**Figure 1:**
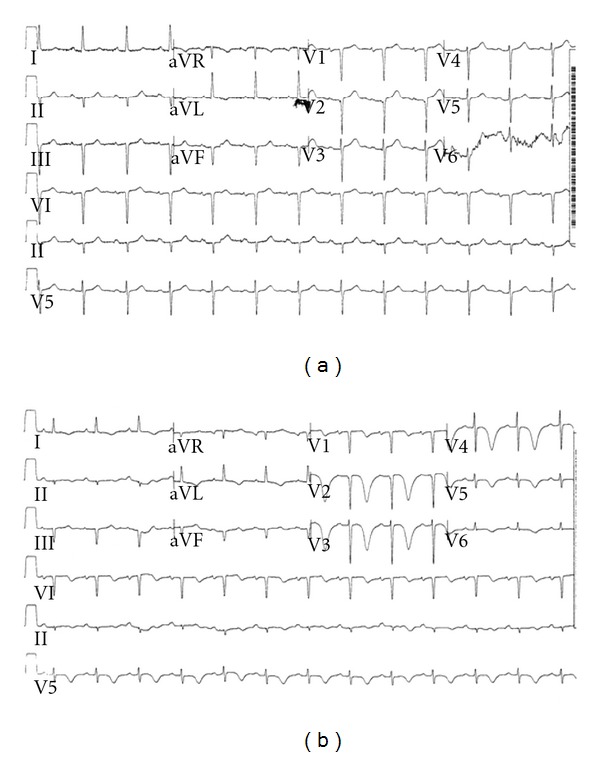
(a) ECG on presentation. (b) ECG performed on the next morning showing symmetrical giant precordial T wave inversion with prolonged QT interval.

The patient's chest X-ray was normal, and her initial ECG showed sinus rhythm with left axis deviation and no ischemic changes (Figure 1(a)). CT of the head was ordered to exclude intracranial pathology as a cause for the dizziness and vomiting. The CT was unremarkable. By that time (12 hours after presentation) the laboratory reported that a repeated troponin T level was 0.034 ng/mL and the patient was admitted to our intensive care unit (ICU) with suspected ACS. A third troponin T level taken 17 hours after presentation was 0.015 ng/mL. Four sequential CPK and liver enzymes levels during the first 48 hours of hospitalization were normal. ECG performed on admission to the ICU showed mild T wave inversion in the precordial leads. Routine ECG performed on the next morning showed symmetrical GPTI with prolonged QT interval (Figure 1(b)). An echocardiogram demonstrated normal size and global systolic function of both ventricles, mild concentric hypertrophy of the left ventricle, and mild mitral regurgitation. 

While a diagnosis of acute myocardial infarction was excluded given the lack of significant elevation and typical fall in troponin T, ACS with anterior wall ischemia was still considered the probable diagnosis given the dynamic ECG changes. The patient was therefore referred for coronary angiography which revealed normal coronary arteries. 

The patient's symptoms resolved spontaneously during the first day of hospitalization, and during the rest of her four-day hospitalization she was asymptomatic. 

She was discharged with a final diagnosis of gastroenteritis, most probably viral. Routine ECG on discharge showed normalization of the inverted T waves in the precordial leads. 

## 3. Discussion

Giant precordial symmetrical T wave inversion (GPTI) on ECG may be the result of several cardiac and noncardiac pathologies. When noted in patients with ACS, GPTI is usually found in patients with significant stenosis of the left anterior descending coronary artery [1]. GPTI and QT prolongation may be found in patients with cardiogenic but nonischemic pulmonary edema after resolution of the symptoms [2].

Acute pulmonary embolism may occasionally result in GPTI and QT prolongation [3], GPTI and QT prolongation may develop following subarachnoid hemorrhage, a finding which is associated with myocardial dysfunction [4].

GPTI may appear postpacing, a phenomenon known as cardiac memory. GPTI on ECG may be seen in patients with apical hypertrophy [5].

When the etiology for GPTI is ACS, pulmonary edema, pulmonary embolism, or subarachnoid hemorrhage, the repolarization abnormality is temporary and T waves will normalize usually within one week while in apical hypertrophy GPTI will be present indefinitely. 

The correct etiology of GPTI in an individual patient can usually be assessed by careful history and physical examination.

In this paper we describe an additional etiology for GPTI. Our patient presented with gastrointestinal symptoms and after exclusion of other potential etiologies for GPTI (i.e., ACS, pulmonary edema, pulmonary embolism, subarachnoid hemorrhage, and apical hypertrophy) as well as for other abdominal disorders (i.e., hepatitis, cholestasis, and pancreatitis) a final diagnosis of gastroenteritis was made. 

The patient's troponin was mildly elevated. It is well documented that many nonischemic medical conditions may result in troponin elevation [6]. In our patient, coronary spasm due to stress caused by gastroenteritis may have resulted in this minor troponin elevation. Myocarditis is another potential etiology causing both troponin elevation and ECG changes. We find this diagnosis very unlikely since our patient had no symptoms or signs to suggest myocarditis (e.g., fever, flu like symptoms, and chest pain). In addition, echocardiography showed normal ventricular function.

Our patient apparently had 2 such episodes of gastroenteritis associated with resolving GPTI over a period of 10 years. 

Clinicians should be aware of the different etiologies of GPTI including that demonstrated in our report in order to expedite appropriate treatment. However, in every case, ACS should be excluded.
